# Signaling Crosstalk between Salicylic Acid and Ethylene/Jasmonate in Plant Defense: Do We Understand What They Are Whispering?

**DOI:** 10.3390/ijms20030671

**Published:** 2019-02-04

**Authors:** Ning Li, Xiao Han, Dan Feng, Deyi Yuan, Li-Jun Huang

**Affiliations:** 1 State Key Laboratory of Cultivation and Protection for Non-Wood Forest Trees, Ministry of Education, Central South University of Forestry and Technology, Changsha 410004, China; nli@csuft.edu.cn (N.L.); yuan-deyi@163.com (D.Y.); 2 College of Biological Science and Engineering, Fuzhou University, Fuzhou 350116, China; hanxiao@caas.cn (X.H.); gygzgzyx@126.com (D.F.); 3 Biotechnology Research Institute, Chinese Academy of Agricultural Science, Beijing 100081, China

**Keywords:** hormones, signaling pathway, plant defense

## Abstract

During their lifetime, plants encounter numerous biotic and abiotic stresses with diverse modes of attack. Phytohormones, including salicylic acid (SA), ethylene (ET), jasmonate (JA), abscisic acid (ABA), auxin (AUX), brassinosteroid (BR), gibberellic acid (GA), cytokinin (CK) and the recently identified strigolactones (SLs), orchestrate effective defense responses by activating defense gene expression. Genetic analysis of the model plant *Arabidopsis thaliana* has advanced our understanding of the function of these hormones. The SA- and ET/JA-mediated signaling pathways were thought to be the backbone of plant immune responses against biotic invaders, whereas ABA, auxin, BR, GA, CK and SL were considered to be involved in the plant immune response through modulating the SA-ET/JA signaling pathways. In general, the SA-mediated defense response plays a central role in local and systemic-acquired resistance (SAR) against biotrophic pathogens, such as *Pseudomonas syringae*, which colonize between the host cells by producing nutrient-absorbing structures while keeping the host alive. The ET/JA-mediated response contributes to the defense against necrotrophic pathogens, such as *Botrytis cinerea*, which invade and kill hosts to extract their nutrients. Increasing evidence indicates that the SA- and ET/JA-mediated defense response pathways are mutually antagonistic.

## 1. Introduction

As sessile organisms, plants are under frequent attack from a broad spectrum of microbial pathogens, including viruses, bacteria, fungi and oomycetes, in their living environments. The pathogens can be classified as either biotrophic or necrotrophic according to their different infection strategies [1]. Biotrophic pathogens first penetrate epidermal cells and multiply in the intercellular spaces by feeding on living host tissue. Most of the biotrophic pathogens are host-specific, such as *Pseudomonas syringae*. Necrotrophic pathogens kill host plant cells using toxic metabolites and then feed on the remains. Most of the necrotrophs infect a wide range of hosts. During evolutionary warfare with pathogens, plants have evolved sophisticated detection and defense systems to ward off pathogen invasion.

The investigation of Arabidopsis mutants with defects in salicylic acid (SA) biosynthesis and signaling pathways for altered pathogen susceptibility has demonstrated that SA is a crucial defense signal molecule against biotrophs [2,3,4]. SA is required for the activation of both pathogen-associated molecular patterns (PAMPs)-triggered immunity (PTI) and effector-triggered immunity (ETI). Parallel approaches have demonstrated that phytohormones, ethylene and jasmonate, play a major role in defense responses against necrotrophs. The Arabidopsis jasmonate- or ethylene-insensitive mutants display enhanced susceptibility to the necrotrophic *Botrytis cinerea*. Those mutants have no effect on resistance to biotrophs [5,6]. The infection of Arabidopsis plants with biotrophic *P. syringae*, which triggers the SA-mediated defense response, results in significantly compromised resistance against necrotrophs by suppression of the jasmonate/ethylene (ET/JA) signaling pathway [7]. This experiment demonstrated the existence of crosstalk between SA and ET/JA signaling pathways. A considerable work using molecular, biochemical and genomic tools has been carried out to decipher the underlying mechanism. So far, the crosstalk has been found to occur at multilayers of regulation, including phytohormone metabolism, gene expression and protein modification [8]. As is often the case with understandable, reasonable explanations, the real situation is more convoluted. Therefore, more investigation and discussion are needed.

The classical phytohormones, such as abscisic acid (ABA), auxin, brassinosteroid (BR) and cytokinin (CK) were adopted to fine-tune the plant defense response. The roles and models of those chemicals have been comprehensively discussed in many reviews [9,10,11,12,13] and are beyond the scope of this review. Here, we first compile recent progress in the biosynthesis regulation and signaling pathway of those defense hormones. We then discuss and explore the most up-to-date understanding of the signaling crosstalk, with particular emphasis on transcriptional regulation.

## 2. SA-Mediated Defense Signaling Pathway against Biotrophic Pathogens

SA is a phenolic compound that has been shown to regulate various aspects of plant growth and development. SA is also a critical signaling molecule for activating defense responses against pathogen infection. The first indication of the involvement of SA in pathogen responses was provided by White et al. [14]. They showed that the injection of SA into tobacco leaves led to pathogenesis-related (PR) protein production and increased resistance to tobacco mosaic virus (TMV). Since then, SA has been shown to induce *PR* gene expression and enhance resistance in a broad spectrum of plant species [15]. Conclusive evidence supporting SA as a critical signal in the defense response was produced from studies using Arabidopsis. Plants with reduced SA amounts due to the ectopic expression of the bacterial *nahG* gene (a SA-degrading salicylate hydroxylase) or dysfunction of the SA biosynthesis *SID2/ICS1* gene (*salicylic acid induction deficient 2*/*isochorismate synthase 1*) exhibited reduced local and systemic resistance and were more susceptible to biotrophic pathogen infection, whereas the exogenous application of SA restored the resistance [2,3].

### 2.1. SA Biosynthesis and Regulation

**Figure 1:**
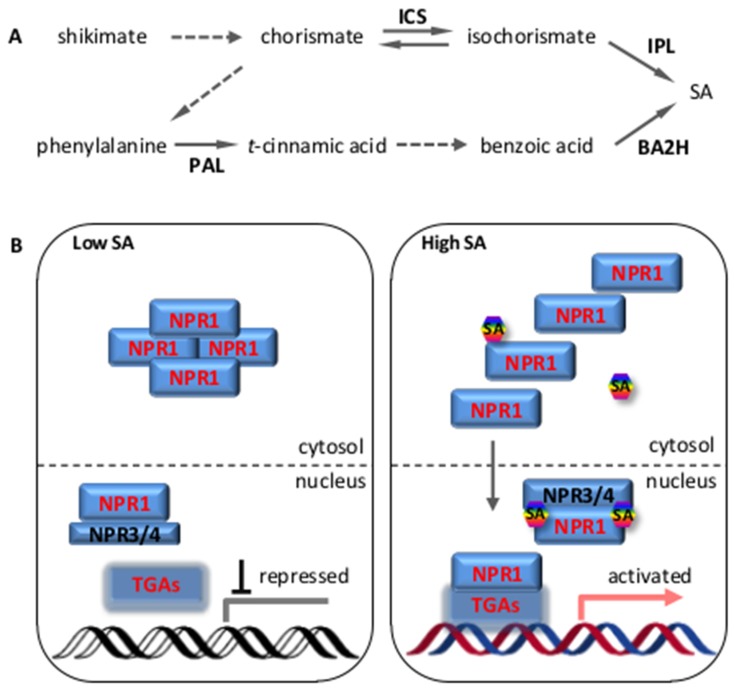
Salicylic acid biosynthesis and signaling pathway. (**A**) Proposed model for salicylic acid (SA) biosynthesis in Arabidopsis. Upper panel: the isochorismate pathway revealed by genetic studies. Lower panel: the phenylpropanoid pathway revealed by biochemical studies. (**B**) A simplified model for the SA signaling pathway according to Ding et al. [18] and Mou et al. [19]. In cells with low SA levels, NPR1 forms oligomer and remains in the cytosol, NPR3 and NPR4 bind residual NPR1 in the nucleus to prevent NPR1 function. In cell with high SA levels, NPR1 becomes monomeric and enters the nucleus, where SA binds to NPR3 and NPR4 to block their transcriptional repression activity. NPR1 interacts with TGAs in SA-responsive promoters, leading to the activation of defense responses. Abbreviations: BA2H, benzoic acid 2-hydroxylase; ICS, isochorismate synthase; IPL, isochorismate pyruvate lyase; NPR, non-expresser of pathogenesis-related genes; PAL, phenylalanine ammonia lyase; SA, salicylic acid; TGA, TGACG-binding factor.

Recent characterization of the SA biosynthetic pathway revealed two distinct branches (Figure 1A)—the isochorismate pathway and the phenylpropanoid pathway—but both branches require the chemical chorismate derived from the shikimate pathway.

As revealed by early biochemical feeding studies with radio-labelled substrates and specific enzyme inhibitors (i.e., 2-aminoindan-2-phosphonic acid), the phenylpropanoid pathway for SA biosynthesis begins with the conversion of phenylalanine (Phe) to *trans*-cinnamic acid (*t*-CA), which is catalyzed by phenylalanine ammonia lyase (PAL). *t*-CA is then converted to benzoic acid (BA), for which the enzyme responsible is not yet known. SA is subsequently produced from BA via hydroxylation, which is catalyzed by benzoic acid 2-hydroxylase (BA2H) (Figure 1A). However, genetic studies indicated that the parallel isochorismate pathway accounts for the majority of pathogen-induced SA accumulation. Two Arabidopsis mutants, *sid2-1* and *eds16-1* (*enhanced disease susceptibility 16-1*), which exhibited only 5–10% of the wild-type level of SA upon pathogen challenge, were both found to contain a lesion in the *ICS1* gene [3,16]. The isochorismate pathway occurs in the plastids. First, the enzyme ICS1 converts chorismate to isochorismate and isochorismate is then converted to SA by isochorismate pyruvate lyase (IPL) (Figure 1A). Arabidopsis contains two ICS genes: *ICS1* and *ICS2*. The residual amount of SA in the pathogen-infected *ics1* (*sid2-1*/*eds16-1*) mutant might be synthesized by ICS2 or might originate from the phenylpropanoid pathway. However, the Arabidopsis *IPL* gene is still not characterized; thus, the SA biosynthetic pathway has not been fully elucidated. Most recently, Zhou et al. reported the isolation of an Arabidopsis peroxidase encoded by *PRXR1*, which might have the IPL enzyme activity. PRXR1 facilitate the conversion of isochorismate to SA when expressed in *E. coli* [17].

Studies have shown that *ICS1* is locally and systemically induced during pathogen infection [3]. Several transcription factors have been isolated that regulate *ICS1* expression. Zhang et al. identified plant-specific transcription factors—SARD1 (SAR-deficient 1) and CBPg60 (calmodulin-binding protein 60-like g)—which both bind to the *ICS1* promoter and regulate the induction of *ICS1* expression [20]. van Verk et al. showed that WRKY28, of the WRKY transcription factor family, binds to two W-box motifs in the *ICS1* promoter and activates the *ICS1* promoter in a protoplast transient expression assay, suggesting that WRKY28 might be a positive regulator of *ICS1* expression [21]. In addition to these positive transcription activators, EIN3 (Ethylene Insensitive 3) and ANAC019 and their homologs were shown to serve as repressors of *ICS1* expression [22,23]. These genes are positive regulators of ET- and JA-signaling pathways, indicating the possible crosstalk between these hormones. It has been speculated that a negative feedback loop for SA biosynthesis exists [24]. The induction of *ICS1* leads to SA accumulation and SA activates NPR1 (non-expresser of pathogenesis-related genes 1), a master regulator of downstream SA signaling. Besides activating SA-responsive genes, NPR1 also acts as a negative regulator of *ICS1* gene expression [24], thereby closing the negative feedback loop. Upon bacterial pathogen infection, the *npr1* plants accumulated significantly higher levels of *ICS1* transcripts and free SA than the wild-type plants. The molecular mechanism through which NPR1 represses *ICS1* promoter is unclear. NPR1 might induce members of the WRKY transcription factors with a transcriptional repressive activity to suppress *ICS1* expression and to prevent SA content from elevating to escalating [24].

As a defense signal, SA levels are tightly controlled in plants. In addition to regulation at the biosynthesis level, SA is regulated through metabolism. For instance, free SA undergoes a variety of chemical modifications including glycosylation, methylation and amino acid conjugation. SA is glucosylated by SA glucosyltransferase (SAGT) to form the inactive SA-glucoside (SAG), which allows the vacuolar storage of less toxic SA-glucoside in relatively large quantities. The methylation of SA catalyzed by BA/SA carboxyl methyltransferase 1 (BSMT) leads to the formation of methyl salicylate (MeSA). Park et al. suggested that this volatile MeSA served as a systemic signal for SAR [25].

### 2.2. SA Signaling Transduction through NPR and TGA

A considerable body of work, mainly from the Dong group, proved that NPR1 (also known as NIM1) is a master regulator of the SA-mediated defense signaling. The activity of NPR1 is mostly controlled at the post-transcriptional level. Recent studies showed that SA directly binds to NPR1 and NPR1 homologs and possibly regulates NPR1 stability and activity [26,27]. Mou et al. found that increased cellular SA levels trigger a redox change in the cytoplasm that switches NPR1 from the oligomer to monomer forms [19] (Figure 1B). The monomerization is catalyzed by thioredoxins TRX-h3 and TRX-h5 via the reduction of a cysteine residue (Cys156). The active monomers then translocate to the nucleus and work together with other transcription factors to activate SA-responsive gene expression. In the resting cells, Tada et al. showed that S-nitrosoglutathione (SNO) promotes NPR1 oligomer formation via the S-nitrosylation of Cys156 [28]. Spoel et al. revealed that, in the nucleus, the NPR1 ubiquitination mediated by the Cullin3 (CUL3) E3 ligase and degradation by the 26S proteasome are required for the full induction of the NPR1 target genes [29]. SA also triggers the phosphorylation of NPR1 at serine residues 11 and 15 (Ser11 and Ser15, respectively), which facilitates NPR1 interaction with CUL3 and promotes turnover of NPR1. Spoel et al. hypothesized that the degradation and de novo synthesis of active NPR1 was a prerequisite for each round of transcription [29]. Saleh et al. reported that NPR1 is phosphorylated at serine residues 55 and 59 in the resting cells and associates with transcriptional repressors to silence the SA-responsive gene [30]. SA accumulation triggers the dephosphorylation of Ser55/Ser59 and sumoylation at the SIM3 domain and this modification promotes the phosphorylation of Ser11/Ser15. The active form of NPR1 interacts with transcriptional activators of the TGACG-binding factor (TGA) transcription factor family to induce gene expression. Consistent with these findings, sumoylation-deficient NPR1 leads to compromised local- and systemic-acquired resistance [30].

Fu et al. demonstrated that NPR3 and NPR4, which exhibit different binding affinities toward SA, are the long-sought-after nuclear receptors of SA [26]. In uninfected plants, basal SA is sensed by the low binding affinity receptor NPR4, which interacts with NPR1 and this results in to NPR1 degradation. In infected plants, a high concentration of SA induces NPR3 and NPR1 interaction, which leads to turnover of NPR1 and defense-associated programmed cell death at the site of infection (local part). At the uninfected distal site (systemic part), a high level of SA induces the activation of NPR1 and the expression of defense genes. It was postulated that NPR1 acts as a SA receptor and binding to SA seems to be required for the full disassembling of the NPR1 oligomer to monomer forms [27].

Since NPR1 contains only a transactivation domain but no DNA-binding domain, NPR1 exerts its transcriptional activity through interaction with other transcription factors. Members of the TGA family of basic leucine zipper (bZIP) transcription factors interact with NPR1 in yeast-two-hybrid and transient *in planta* assays [31]. In the Arabidopsis genome, 10 members of the TGA family are found, which are further divided into five sub-clades: clade I contains TGA1 and TGA4; clade II contains TGA2, TGA5 and TGA6; clade III includes TGA3 and TGA7; clade IV includes TGA9 and TGA10; and clade V contains only PAN (TGA8). The clade II (TGA2, 5 and 6) and III (TGA3 and 7) members interact with NPR1 constitutively in yeast cells. The interaction of NPR1 with the clade I members (TGA1 and 4) was only found in SA-stimulated leaves. These TGAs differentially bind to both positive and negative *cis-elements* in the *PR1* promoter. Genetic analysis revealed distinct and redundant roles of TGAs on basal and acquired resistance in terms of *PR1* expression [32]. Upon SA treatment, the binding of TGA2 and TGA3 to the *PR1* promoter is enhanced by interaction with NPR1 [33,34].

In addition to TGAs, NIMINs (NIM1-interacting) also interact with NPR1 and operate as negative regulators. All three members of the NIMINs (NIMIN1, 2 and 3) contain a transcriptional repressor motif EAR (Ethylene-responsive element binding factor-associated amphiphilic repression), which functions as an adaptor to recruit the corepressor TOPLESS (TPL). However, it has not yet been characterized whether TPL is involved in inhibiting NPR1-TGA-mediated *PR1* expression [35,36]. Most recently, Ding et al. reported that NPR3 and NPR4 function redundantly as transcriptional co-repressors and their function is inhibited in the presence of SA [18] (Figure 1B). Therefore, SA regulates NPR1 activity at different levels: (1) SA-induced redox changes lead to NPR1 monomerization and nuclear translocation [19,28], (2) SA-triggered post-transcriptional modifications regulate NPR1 degradation and transcriptional activity [29,30] and (3) SA binding to NPR1 modulates its abundance and transcriptional co-activator function [18,26,27].

## 3. ET/JA-Mediated Defense against Necrotrophic Pathogens and Signaling Pathways

Both the ethylene (ET) and jasmonic acid (JA) signaling pathways are required for the activation of plant defense against necrotrophic pathogens. In either ET- or JA-insensitive mutants, the induction of pathogen defense genes (i.e., *PDF1.2*) is drastically reduced [37].

### 3.1. ET Biosynthesis and Signaling Pathway

**Figure 2:**
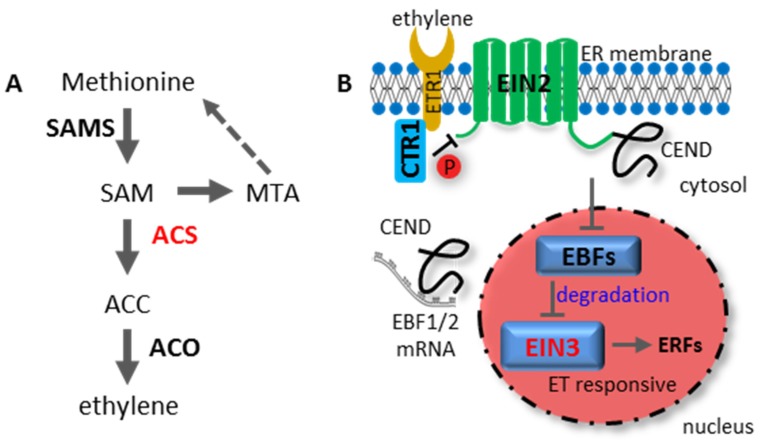
Ethylene (ET) biosynthesis and the signaling cascade pathway. (**A**) Model for the ET biosynthesis pathway. The precursor SAM is produced by SAMS with methionine as substrate. SAM is converted to the intermediate chemical ACC by ACS with the release of MTA as byproduct. MTA is recycled to methionine through the so-called Yang cycle. The rate-limiting enzyme ACS is highlighted in red. (**B**) Model for the ET signaling cascade. In the absence of ET, CTR1 phosphorylates EIN2 and the ET pathway is therefore blocked. In the presence of ET and when it is perceived by ET receptor (i.e., ETR1, ETHYLENE RESISTANT 1), the kinase activity of CTR1 is inactivated, the EIN2 CEND becomes dephosphorylated and cleaved. CEND subsequently translocates into the nucleus to attenuate EBFs E3 ligase function. In addition, CEND may bind to the UTR of EBF1/2 mRNA to perturb EBF1/2 translation in cytosol. Stabilized EIN3 protein then activates ERF transcription factors (i.e., ERF1 and ORA59) to elicit the ET response. Abbreviations: ACC, 1-Aminocyclopropane-1-carboxylic acid; ACO, ACC-oxidase; ACS, ACC synthase; CEND, C-terminal end of EIN2; CTR1, constitutive triple response 1; EBF1/2, EIN3-binding F-Box 1/2; EIN, ethylene insensitive; ER, endoplasmic reticulum; ERF, ethylene-response factor; ET, ethylene; ETR1: ethylene-resistant 1; MTA, methylthioadenosine; SAM, S-adenosyl methionine; SAMS, SAM synthase.

ET is a gaseous hormone that has been recognized as a plant growth regulator for more than a century. The ET biosynthetic pathway, also known as the Yang cycle, begins with the amino acid methionine [38] (Figure 2A). 1-aminocyclopropane-1-carboxylic acid (ACC) synthase (ACS) is a rate-limiting enzyme of ET biosynthesis, which converts S-adenosyl methionine (SAM) to ACC [39]. As a diffusible, gaseous and non-degradable hormone, ET biosynthesis has to be tightly controlled. Therefore, the regulation of ACS activity confers strict control of ET production.

After accumulation, ET is perceived by endoplasmic reticulum (ER)-localized receptors, which act as negative regulators of the ET signaling pathway [40]. Upon ET binding, the ER-localized EIN2 become dephosphorylated due to the inactivation of the Raf-like kinase CONSTITUTIVE TRIPLE RESPONSE 1 (CTR1) associated with the receptors. Dephosphorylated EIN2 releases its C-terminal domain (CEND), which enters into the nucleus and conveys signals to the EIN3 transcription factor [41,42,43]. EIN3 directly activates the expression of an array of ET-responsive transcription factors such as *ETHYLENE RESPONSE FACTOR 1 (ERF1)* and *OCTADECANOID-RESPONSIVE ARABIDOPSIS AP2/ERF 59 (ORA59)*, which magnify and elicit the ET response [44,45]. ET also stabilizes EIN3 protein by eliminating two F-box proteins, EIN3 BINDING F-BOX PROTEIN 1 (EBF1) and EBF2, which target EIN3 for proteasomal degradation in the absence of ET [46]. The expression of EBF1 and EBF2 are induced by EIN3, providing a negative feedback loop for the ET signaling pathway [47]. Both Li et al. and Merchante et al. discovered that ET-released C-terminal portion of EIN2 directly bound to 3′UTR of EBF1/2 mRNA for translational repression [48,49] (Figure 2B).

### 3.2. JA Biosynthesis and Signaling Pathway

Jasmonate (JA) and its derivatives are oxygenated-lipids (oxylipins)-based hormones that play important roles in the regulation of plant defense and development [50].

**Figure 3:**
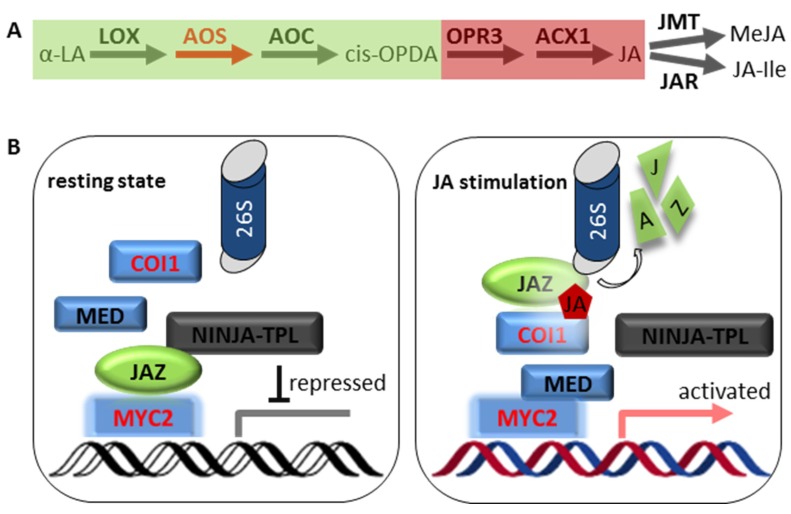
Jasmonate (JA) biosynthesis and signaling transduction pathway. (**A**) Model for the JA biosynthesis pathway. The intermediate OPDA is synthesized in the chloroplasts. JA is synthesized in the peroxisomes and exported to the cytosol, where it is converted to other bioactive derivates (i.e., JA-Ile). The key enzyme AOS is highlighted in red. (**B**) Model for the JA signaling transduction pathway of the MYC-branch in Arabidopsis. In the non-induced cells (left, low JA level), MYC2 activity is repressed by JAZ proteins that interact with NINJA to recruit transcriptional repressor TPL. In the JA-stimulated cell (right, high JA level), JAZ proteins are degraded by the SCF^COI1^-mediated 26S-proteosome. MYC2 is released to interact with the transcriptional mediator to activate JA-responsive gene expression. Abbreviations: α-LA, α-linolenic acid; ACX1, acyl-CoA-oxidase 1; AOC, allene oxide cyclase; AOS, allene oxide synthase; COI1, coronatine insensitive 1; JA, jasmonic acid; JA-Ile, Jasmonic acid-isoleucine conjugate; JAR, jasmonate resistant; JAZ, jasmonate ZIM domain; JMT, JA methyl transferase; LOX, 13-lipoxygenase; MeJA, methyl jasmonate; MED, mediator; NINJA, novel interactor of JAZ; OPDA, 12-oxophytodienoic; OPR3, OPDA Reductase 3; TPL, TOPLESS.

The biosynthesis of JA starts with the oxygenation of the lipid substrate, linolenic acid (18:3), in chloroplasts (Figure 3A). The end product of a series of reactions catalyzed by 13-lipoxygenase (LOX), allene oxide synthase (AOS) and allene oxide cyclase (AOC) is 12-oxophytodienoic (OPDA). In Arabidopsis, ghd mutation of the *AOS* gene results in a complete loss of JA production. OPDA produced in chloroplasts is transported into peroxisomes, where it is subsequently reduced by OPDA Reductase 3 (OPR3) and oxidized by acyl-CoA-oxidase 1 (ACX1) resulting in JA formation. The genes participating in JA synthesis are inducible by JA, thus providing a positive feedback loop. JA produced in peroxisomes is transported to the cytosol.

To control the activity of JA in plants, it undergoes differential modifications, for instance, JA hydroxylation, decarboxylation, glycosylation, methylation catalyzed by a JA methyl transferase (JMT) and amino acid conjugation by a JA conjugate synthase (JAR1, jasmonate resistant 1). JA-Ile, produced by JAR1, is the final biological active compound in plants [51,52]. JA-Ile was identified as the ligand of jasmonate receptor complex, consisting of Coronatine Insensitive 1 (COI1), JA-Ile and member of the Jasmonate ZIM Domain (JAZ) proteins [53,54].

A major breakthrough in understanding the JA signaling pathway was the isolation of JAZ proteins, which were later found to be components of the JA co-receptors [55]. JAZs are suppressors of JA-induced transcriptional response. In the absence of JA, JAZ proteins recruit the transcriptional co-repressor TPL via interaction with the bridging protein Novel Interactor of JAZ (NINJA) [56]. Upon stress, accumulated JA-Ile binds to the F-box protein COI1 to facilitate the formation of COI1-JAZs complex, resulting in ubiquitination and the ultimate degradation of JAZ repressors via the 26S proteasome [57,58]. Downstream of COI1-JAZ perception, the JA signaling pathway can be divided into two distinct branches: the MYC-branch and the ERF-branch [8].

The MYC-branch is mainly responsible for wounding- and insect-induced JA signaling pathway. This branch is controlled by the basic helix-loop-helix leucine zipper transcription factors MYC2, MYC3 and MYC4. In the absence of JA, JAZ repressors interact with MYC proteins and recruit the co-repressor TPL (Figure 3B). A recent study by Zhang et al. showed that the JAZ interaction with MYC protein competitively block their interaction with the MED25 subunit of the transcriptional Mediator complex [59]. Activation of MYC-branch upon removal of JAZs leads to expression of a large set of JA-responsive genes, including JA marker gene *VSP2*, JA synthesis gene *LOX2* and JA signaling repressor *JAZ* genes. 

The ERF-branch is induced upon necrotrophic pathogen infection. This branch is synergistically regulated by the ET-signaling pathway and controlled by the AP2/ERF-Domain transcription factors ORA59 and ERF1, which directly activate the expression of ERF-branch marker genes, like *PDF1.2*. ORA59 and ERF1 specifically bind to the GCC-box motif via the ERF domain. The GCC-boxes are essential for activation of *PDF1.2* expression [44]. However, whether JAZ repressors interact directly or indirectly with ERFs is unknown. Zhu et al. reported that JAZ proteins directly interacts with EIN3 and represses EIN3 induced *ORA59* and *ERF1* expression [60]. Within the JA responsive pathway, the MYC- and ERF-branches are mutually antagonistic.

## 4. Signaling Crosstalk between SA- and ET/JA-Mediated Pathways

Plant defense responses against environmental pathogens are energy consuming. Ideally, plants employ a specific pathway upon recognizing of distinct pathogens. Extensive crosstalk between different signaling pathways provides the potential for efficient energy allocation. For instance, the SA- and ET/JA-mediated defense signaling pathways act in both synergistically and antagonistically. Treatment with low concentrations of SA and JA has been reported to result in synergistic expression of both the SA target gene *PR1* and the JA marker gene *PDF1.2*, whereas higher concentrations of SA and JA produce the antagonistic expression of these genes [61]. Here, we mainly focus on the antagonistic effect of these pathways.

### 4.1. SA Inhibits ET/JA-Signaling Downstream of JA Biosynthesis

Much attention has been paid to the SA-mediated antagonistic effect on the ET/JA-pathway. Infection with the biotrophic pathogen *Pseudomonas syringae*, which induces the SA pathway, leads to increased susceptibility to the necrotrophic pathogen *Alternaria brassicae* in host plants due to the repression of the ET/JA-pathway [7].

However, the molecular mechanism through which SA antagonizes the ET/JA-signaling pathway is largely unclear and is a matter under debate. Leon-Reyes et al. reported that SA repression of the JA-signaling pathway is independent of JA biosynthesis [62]. Although a list of JA biosynthesis genes, such as *LOX2*, *AOS*, *AOC2* and *OPR3*, are repressed by SA, the authors demonstrated that the exogenous application of SA represses JA-induced marker gene *PDF1.2* expression to the same level in the *aos* mutant as in the wild-type (WT) plants. The repression occurred downstream of JA perception. In the JA-receptor *coi1* mutant, the induction of *PDF1.2* by ERF1 or ORA59 was also repressed by SA [62].

### 4.2. SA Antagonizes the ET/JA-Signaling Pathway at the Gene Transcriptional Level

Recent studies suggest that repression of the ET/JA-signaling pathway by SA is mainly controlled at the gene transcription level. The SA-signaling pathway induces negative regulators to interfere with the ET/JA-regulated transcription factors of the ERF branch.

Li et al. reported that the ectopic expression of SA-induced WRKY transcription factor WRKY70 suppresses JA-induced *PDF1.2* expression [63,64]. The WRKY binding site, the W-box motif, is overrepresented in the promoters of SA-repressed ET/JA-responsive genes. Thus, SA-induced WRKY70 may inhibit the ET/JA-responsive gene expression via directly binding to the promoters. However, SA still actively represses JA-responsive marker gene *PDF1.2* expression in *wrky70* knock-out mutants. This result indicates either the functional redundancy of different WRKYs or that *WRKY70* is only sufficient but not necessary for SA-ET/JA crosstalk. It is an interesting issue needs further clarification.

As introduced above, the TGA transcription factors (TGA2, TGA5 and TGA6) are positive regulators of the NPR1-dependent SA-signaling pathway. The clade II TGA TFs have both positive and negative roles in the ET/JA-signaling pathway. Zander et al. showed that the induction of *PDF1.2* is blocked in the young axenic-cultured triple mutant after a combination of ACC (an ET precursor) and JA treatments. The expression of *PDF1.2* is not induced upon ACC-treatment or necrotrophic pathogen infection in the adult soil-grown *tga256* mutant plants [65]. In addition, these TGAs are required for the SA-mediated repression of the ET/JA-response. Since the *myc2* mutant showed a hyper-induction of *PDF1.2* expression in young axenic cultured plants after a combination of ACC and JA treatment, ET/JA-induced *PDF1.2* expression is not repressed by SA in the *myc2 tga256* mutants. Similarly, JA-induced *PDF1.2* expression is not repressed by SA in the adult soil-grown *tga2356* mutant [66].

Microarray analysis revealed that approximately 36% (136/374) of ACC-responsive genes are TGA dependent and half (63/136) of these genes, which are induced by ET in a TGAs-dependent manner, are SA targets in the SA-ET/JA crosstalk. According to the microarray analysis, Zander et al. concluded that the master regulator, ORA59, of the ERF-branch is a promising candidate target of TGAs [67]. ACC-induced *ORA59* expression was significantly impaired in *tga256* mutant plants. Subsequently, the authors showed that TGAs directly bind to the *ORA59* promoter in a chromatin immunoprecipitation (ChIP) assay and the binding activity is enhanced by ET. Thus, the targeting of TGAs to the *ORA59* promoter provides an essential regulatory node for the activation and SA-antagonism of ET/JA-responsive genes. Therefore, SA may manipulate the transcriptional activity of the clade II TGAs to control the ET/JA-signaling pathway. This idea became more plausible after the identification of an SA-inducible plant-specific glutaredoxin (*GRX*) *ROXY19* (also known as *GRX480* or *GRXC9*) in Arabidopsis, which physically interacts with the clade II TGA factors in yeast-two-hybrid assays. The ectopic expression of *ROXY19* strongly represses the ET/JA-induced *ORA59* and *PDF1.2* expression in a clade II TGA-dependent manner [68]. GRXs are ubiquitous small redox enzymes that maintain a cellular redox state [69]. It has not been determined if those TGAs are direct substrates of ROXY19.

However, van der Does et al. reported that the GCC-box is sufficient for the SA-mediated repression of ET/JA-responsive gene expression and the crosstalk occurs at a level downstream of *ORA59* gene expression. They found that the ORA59 protein is eliminated upon SA application [70]. This result suggests that SA represses the ET/JA-signaling pathway through the degradation of a transcriptional activator.

The master regulator NPR1 has been identified as an essential integrator for SA-ET/JA crosstalk. NPR1 is at least required for SA-induced *WRKY70* and *ROXY19* expression, which are employed to repress the ET/JA-signaling pathway. Spoel et al. showed that, in *npr1* mutant plants, the repression effect of SA on JA-induced *PDF1.2* expression is completely abolished [71]. Surprisingly, the translocation of NPR1 from the cytosol to the nucleus after switching from the oligomer to monomer the form, which is critical for SA-mediated response, is not required for SA-ET/JA crosstalk. The expression of a chimeric NPR1 protein that is retained in the cytosol was shown to be sufficient to repress JA-induced *PDF1.2* expression upon SA application. As for the molecular mechanism, it is still unknown how a cytosolic NPR1 exerts its function to mediate the crosstalk.

### 4.3. Possible Role of Epigenetic Regulation during Crosstalk

The hormone SA influences the expression of approximately 10% of the Arabidopsis transcriptome, with such a broad effect indicating a possible association with chromatin remodeling by epigenetic regulation. Chromatin is a dynamic nucleoprotein complex composed of DNA wrapped around histones. Chromatin tightly regulates gene expression by controlling access of regulatory proteins and transcriptional machinery to DNA. Transcriptional activators typically recruit enzymes to modify chromatin structure through the methylation, acetylation, sumoylation and phosphorylation of histone tails. Histone acetyltransferases (HAT) and deacetylases (HDA) are responsible for histone acetylation. In Arabidopsis, the JA- and ET-inducible HDA6 and HDA19 were reported to be involved in regulating the ET/JA-signaling pathway. HDA6 is recruited to repress EIN3-mediated transcription of the ERF-branch via association with the bridging protein JAZs [60]. Conversely, HDA19 is a positive regulator of the ERF-branch and overexpression of *HDA19* confers a plant with more resistance to the necrotrophic pathogen *Alternaria brassicicola* [72]. Therefore, SA may take control of ET/JA-signaling by manipulating the activity of these enzymes. Using pharmacological treatment and ChIP analysis, Koornneef et al. revealed that histone modification at the *PDF1.2* promoter is not altered by SA, indicating that chromatin remodeling is not essential for the crosstalk [73].

### 4.4. JA Negatively Regulates SA Biosynthesis

The activation of the ET/JA-pathway represses the SA response. The deletion of the JA receptor COI1 and JA-responsive MYC branch both result in the increased accumulation of the SA level and enhanced resistance to the biotrophic pathogen *Pseudomonas* *syringae* [74]. Pathogens could manipulate this crosstalk for their own benefits. To promote virulence, the bacterial *Pseudomonas* secrete phytotoxin coronatine (COR), which acts as a JA-Ile mimic and binds to the JA co-receptor COI1-JAZs with high affinity to trigger the JA response [75,76]. At the molecular level, Zheng et al. showed that COR activates the expression of three members of the NAC transcription factor family: *ANAC019*, *ANAC055* and *ANAC072* through the MYC branch. These NAC transcription factors directly repress *ICS1* and activate *BSMT1*, leading to reduced SA accumulation [21].

## 5. Perspectives: Developing Better Defensive Plants via Deciphering Crosstalk

**Figure 4:**
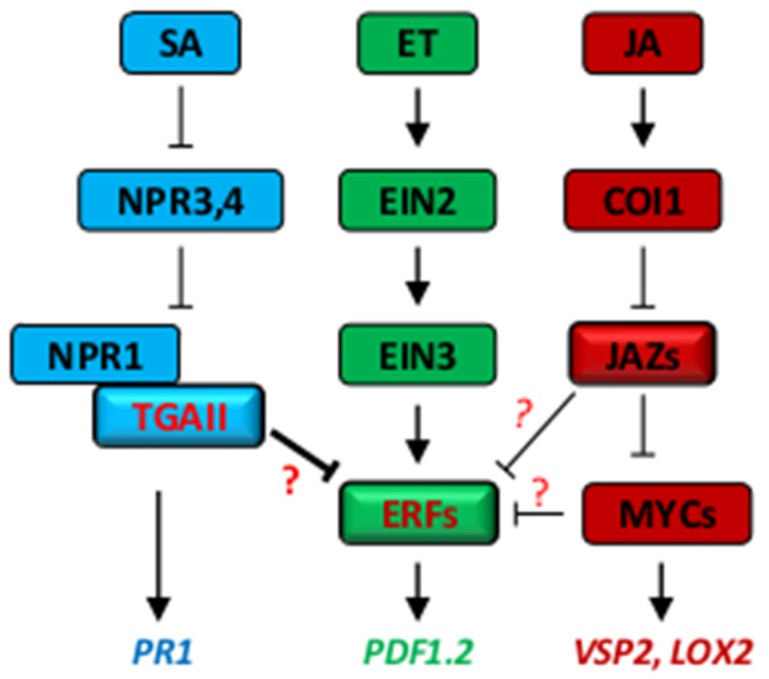
A simplified schematic representation of the signaling network between defense hormones, highlighting the crosstalk at the transcriptional level. Arrows indicate positive effects (activation), blunt-ended lines indicate negative effects (repression), questions indicate unknown mechanisms underlying the repression of ERFs by TGAs, JAZs and MYCs.

So far, the metabolism and signaling transduction of SA, ET and JA have been well elucidated. But, do we fully understand the signaling crosstalk between those hormones? Probably not. In nature, defense hormones work together to manage invading pathogens in an ecological context. However, the interplay between these small molecules has been largely obscured [77,78,79] (Figure 4). 

Due to the high level of complexity, the mechanism that underlies the crosstalk is poorly understood and requires further study. We envision that the newly emerged large-scale OMIC tools and high throughput bioinformatic analysis will be used to seek a better understanding of the crosstalk between these defense hormones, which will ultimately lead to the development of pathogen-resilient crop plants with important agronomical perspectives. For instance, if the crosstalk between SA- and ET/JA-signaling pathways is disconnected, plants will be able to defend against simultaneously colonized biotrophic and necrotrophic pathogens without tradeoffs. Under such conditions, both SA- and ET/JA-signaling pathways are fully armed to fight against corresponding enemies. As the connection node that mediates the crosstalk is still unknown, the gene-piling CRISPR/cas technology serves no purpose. It would be fascinating if, by genetic engineering, the SA-mediated signaling pathway could be rewired to control the ET/JA-signaling pathway. Early biotroph infection will prime the plants for potential necrotrophs in the environment. Interestingly, the SA receptor NPR3/4 has been shown to activate the JA-signaling pathway by promoting the degradation of the JA transcriptional repressor JAZs [80]. Both positive and negative regulatory factors of the signaling pathways are probable targets to modulate defense hormonal crosstalk. It is a particularly exciting area of study to address the signaling crosstalk between those defense hormones, which bears the promise of developing better plants.
